# Research on escalators used as evacuation stairs under fire scenarios

**DOI:** 10.1038/s41598-023-43784-6

**Published:** 2023-10-07

**Authors:** Chunhua Zhang, Xin Wu, Jinquan Chen, Hai Shen

**Affiliations:** 1 College of Safety Science and Engineering, Liaoning Technical University, Fuxin, China; 2 Jingneng (Xilin Gol) Mining Co. LTD, Abaga Banner of Xilin Gol League Mongolia, Nei Monggol Autonomous Region, China

**Keywords:** Engineering, Mathematics and computing

## Abstract

Shopping malls are crowded areas which makes the evacuation routes unable to meet personnel evacuation needs. Therefore, this paper proposes the idea of using escalators evacuation to increasing evacuation routes. In addition, the effects of escalator fire shutters on the use of escalator evacuation paths, and the efficiency of mall personnel evacuation under different conditions were simulated. The results show that the different states of fire shutters have different effects on the emergency evacuation. When the fire shutter is not lowered, it will result in 1 person not being evacuated to complete. However, when the fire shutter is lowered to 1.8 m from the ground or sprinklers are set, escalators can be used for evacuation routes are verified. And compared to the normal descent of the fire shutter (only the staircase evacuation, 2 people not evacuate completed), the evacuation of people is completed and the efficiency of evacuation is improved. This result can improve the new evacuation model for emergency evacuation plans of shopping malls with escalators.

## Introduction

Shopping mall business area is large and the up and down floor spaces are connected, making fire separation difficult to achieve^1^. Most commodities in the mall are combustible items, and this makes fire spread rapidly. Given the many flammable materials, smoke control measures for years, and dense population, the personnel involved in evacuation activities find it difficult to navigate through evacuation channels, resulting in major fire hazards and casualties. Therefore, scholars have studied mall fires and personnel evacuation mainly in terms of smoke prevention measures, path planning, and access problems. In terms of smoke control measures, Shoshe et al.^2^ used FDS software to simulate and compare shopping centers with and without air curtains installed at key locations. It was observed that when the air curtain jets were discharged at 10 m/s, about 37% of the fire-generated heat was confined to the fire source storage reservoir. Moreover, installation of a two-stage air curtain barrier at the fire source and at the stairs confines 68% of the heat to the stairs. Jia et al.^3^ studied the combination of smoke barrier and mechanical smoke exhaust system under the smoke prevention mode. They found that when the smoke barrier was set at a height of 1 m, the mechanical smoke exhaust port could effectively prevent the spread of high temperature smoke under the double-row setting. In terms of path planning, Sun et al.^4^ used an improved multiscale convolutional neural network algorithm to generate a crowd density map for evaluating the high-density crowd. They established an optimal evacuation route from the location of the crowded area to the exit using an improved ant colony algorithm. Using Beijing Xidan Joy City as an example, we found that this method optimized the evacuation route, reduced the evacuation route inflection points by 25% and shortened the evacuation route length by 10%. In terms of access problems, Han et al.^5^ used Pathfinder software to simulate the evacuation of people from a shopping center during peak hours and proposed the evacuation time under the addition of evacuation channels on the top floor without changing the original building structure as much as possible.The total evacuation time was reduced to 697.5 s. Compared with the original evacuation time of 861.8 s, the total evacuation time decreased by 19%. Zhe Huang^6^ demonstrated that escalators may play an important role in the safe evacuation of people in case of fire.

In terms of escalators used for evacuation activities, Naoko et al.^7^ investigated evacuation efficiency of escalators with different heights and lengths in two modes of stationary and upward movement to obtain the speed of pedestrians evacuating using escalators. There findings provided a foundation for subsequent studies on use the movement speed of escalators during evacuation. Hiroyuki et al.^8^ conducted a numerical simulation of a subway station where escalators were deactivated for evacuation. It was observed that evacuation completion time was approximately half of that of an evacuation using only stairs. Tang Jiale^9^ used pathfinder software to simulate the evacuation time of a commercial complex building using pure stairs versus using escalators for evacuation. The results showed that using escalators for evacuation reduced the evacuation time. Ma Hui et al. ^10^ also used the pathfinder software to solve the optimal escalator and staircase dual-channel coupling mode and showed that the coupled evacuation mode evacuation shortened the evacuation time by 16.24% compared with the single staircase evacuation mode.

In previous studies, the installation of smoke protection measures was found to increase the available safe evacuation time, and path planning as well as increasing the number of passages reduced evacuation time for personnel evacuation. However, compared to adding smoke control measures or changing the original smoke evacuation measures, evacuation can be done by using escalators, thus increasing evacuation routes, and without changing the original facilities of the mall. Previously, the escalator was considered as an emergency evacuation measure and treated as an evacuation path during the entire evacuation process but the evacuation under fire scenario, and the state of the fire shutter at the escalator has not been considered. Therefore, this paper intends to use Pyrosim software to simulate the smoke under four working conditions, i.e., fire shutter at the escalator failure, normal, drop to 1.8 m from the ground, and water curtain instead of the fire shutter. Changes in temperature, CO and visibility at the stairs and escalator locations of the mall were analyzed and the safest time to use the escalator as an auxiliary evacuation staircase was calculated according to the fire state. The Anylogic software was used to simulate the evacuation time for each working condition under the fire scenario, and to compare and analyze the evacuation situation of each working condition to obtain the best working conditions under which the fire shutter at the escalator is most conducive to the evacuation of people. Therefore, a new evacuation model for buildings with escalators is proposed in this study.

## Materials and methods

This paper aimed to determine evacuation time of people under four working conditions; fire shutter failure, normal, drop to 1.8 m on the ground, and setting sprinkler during a fire scenario. Firstly, the model was constructed using Pyrosim's own modeling tool. The model was used to simulate the flue gas transportation in the shopping mall under four working conditions, as well as the changes in temperature, CO and visibility at the escalators and stairs. Based on the time threshold of each evacuation index of human body, the available safe evacuation time of escalator and evacuation staircase was determined. Finally, the Anylogic software was used to simulate the evacuation time of people under fire scenario with fire shutter failure at escalator, normal, fire shutter down to 1.8 m on the ground, and setting sprinkler. By comparing and analyzing evacuation conditions under four working conditions, the best state of the fire roller curtain at the escalator was obtained.

### Mall overview and physical modeling

**Figure 1:**
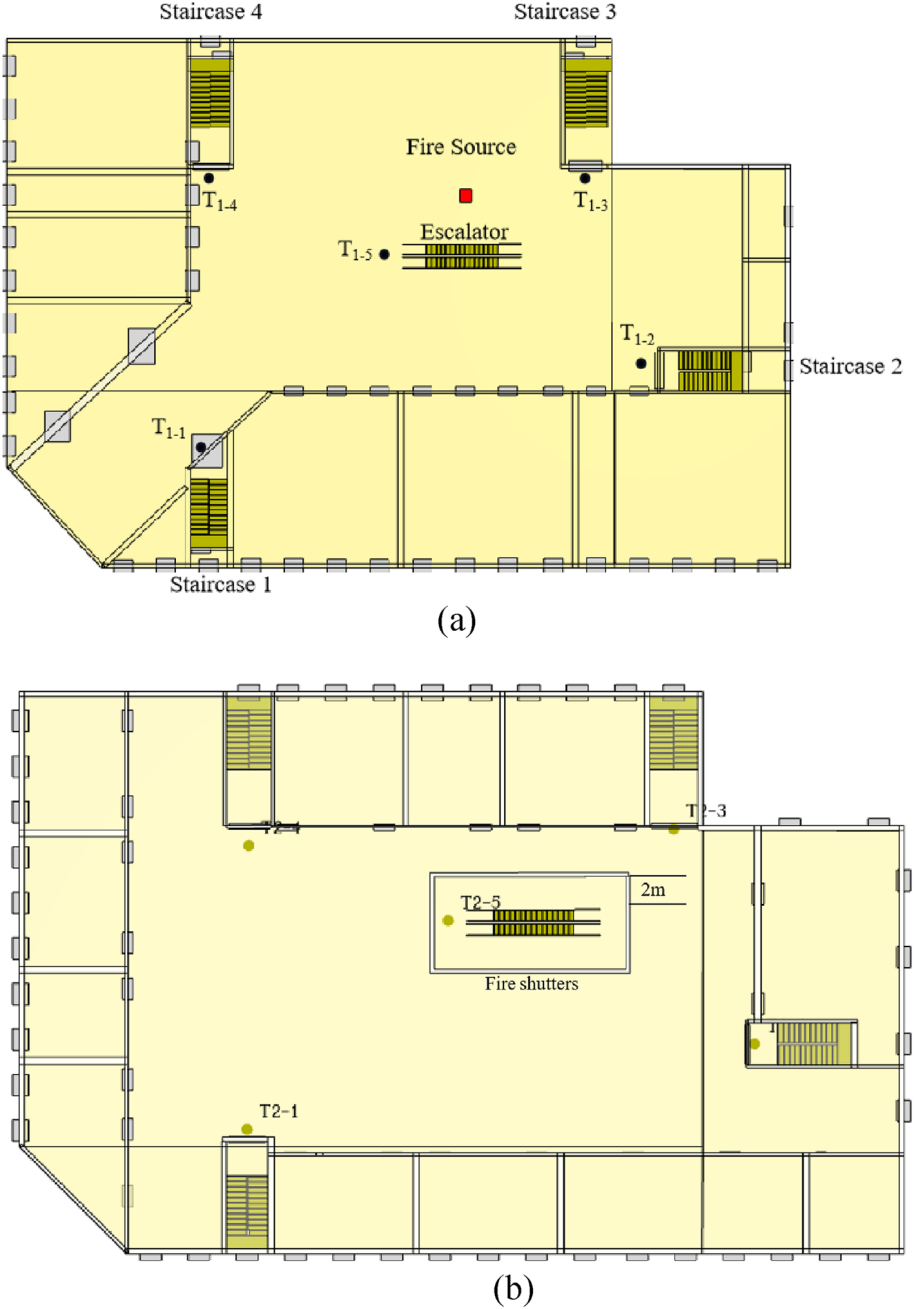
Shopping mall floor plan. (**a**) Floor 1 plan. (**b**) Floor 2, 3 and 4 plans.

The total area of the mall in this study covered a total area of 2620 m^2^, with 4 floors. The first and second floors were commercial centers which mainly contained clothing stores, shopping stores and boutique stores. The third floor provide a children's playground, KTV, leisure and entertainment places. The fourth floor mainly provided catering businesses. One set of escalator was located in the middle of the mall, and four sets of evacuation stairs were distributed around the mall. The net widths of stairs and escalators are 1800 mm and 800 mm respectively. The location of stairs and escalators was shown in Fig. 1. The sprinkler and smoke exhaust facilities in the mall are not well suited to provide safety in case of fire.

### Fire numerical simulation model building

#### Theoretical basis

Pyrosim is a field simulation software, the numerical simulation of fire is based on the principle of Large Eddy Simulation (LES) and follows the basic theory of fluid dynamics (CFD), focusing on the process of calculating the smoke flow and heat transfer under the fire break out scenario^11^. The basic solution equations for the smoke flow, velocity field, temperature field, and concentration field in the simulation are described as follows^2^.

1. Conservation of mass equation
  1 $$\frac{\partial \rho }{{\partial t}} + \nabla \cdot \left( {\rho \vec{u}} \right) = 0$$
  where, *ρ* is the density, kg/m^3^; *t* is the time, s; $\vec{u}$ is the velocity vector, m/s; ∇is the Hamiltonian operator.
2. Conservation of momentum equation
  2 $$\rho \left( {\frac{{\partial \vec{u}}}{\partial t} + \frac{1}{2}\nabla \left| {\vec{u}} \right| - \vec{u} \times \omega } \right) + \nabla P - \rho g = \vec{f} + \nabla \tau$$
  where, $\vec{f}$ is the external force vector acting on the fluid, kg/s^3^/m; *P* is the pressure, Pa;$\tau$ is the velocity viscous force tensor, kg/s^3^/m; g is the acceleration of gravity, m/s^2^; ω is the z-direction velocity, m/s.
3. Conservation of energy equation
  3 $$\frac{\partial }{\partial t}\left( {\rho h} \right) + \nabla \cdot \left( {\rho h\vec{u}} \right) = \frac{\partial \rho }{{\partial t}} + \vec{u} \cdot \nabla \rho - \nabla \mathop {q_{r} }\limits^{ \to } + \nabla \left( {k\nabla T} \right) + \sum\nolimits_{i} {\nabla \left( {h_{i} \rho hD_{i} \nabla Y_{i} } \right)}$$
  where, *i* is the i-th component; Y_i_ is the mass function of the i-th component; *D*_*i*_ is the diffusion coefficient of the i-th component, m^2^/s; mim is the mass production rate of the i-th component, kg/s/m^3^. In Pyrosim, the partial differential equation in the above conservation equation is approximated as finite volume method and solved in real time using a divided three-dimensional grid^12^.

#### Fire growth model

Fire growth models are used to describe the development of a fire. With the exception of explosive fires, most fires show a slow progression from initial to late intense combustion^13^. Such fires can be described by the t^2^ fire model as follows:

4 $$Q_{f} = \alpha t^{2}$$

where *Q*_*f*_ is the exothermic rate of the ignition source, α denotes the fire growth coefficient, and t denotes the burning time. According to the fire model, the fire categories can be classified as slow, medium, fast, and ultra-fast fires, the fire growth coefficient of the fire categories is shown in Table 1. According to the studied model and the characteristics of combustible materials, the fire growth type can be considered as fast, and the fire growth coefficient can be set to 0.044 kW/s^2^.

**Table 1:** The fire growth factor.

Fire category	Typical combustible materials	Fire growth coefficient (kW/s^2^)
Slow fire	Hardwood furniture	0.00278
Medium-speed fire	Cotton, polyester cushions	0.011
Fast fire	Filled mail bags, wooden shelf pallets, foam	0.044
Ultra fast fire	Pool fires, fast-burning decorative furniture, light curtains	0.178

The size of a fire depends on the rate of heat release. The higher the rate of heat release from a fire, the greater the fire hazard. In general, buildings have different rates of heat release from fire sources depending on their characteristics. Therefore, choosing a feasible fire heat release rate in the simulation that reflects most actual fire conditions will result in robust simulation results. The heat release rates for various types of buildings are specified in the Technical Code for Smoke Prevention and Exhaust of Buildings GB51251-2017, the heat release rate of each type of building is shown in Table 2. In this study, the sprinkler system in the mall failed. Considering a realistic worst-case scenario, the fire simulation model in this paper sets the maximum heat release rate to 10 MW.

**Table 2:** Heat release rate of each building.

Building category	Spray or not	Heat release rate *Q* (MW)
Offices, classrooms, guest, rooms, walkways	No	6.0
	Yes	1.5
Shop, exhibition hall	No	10.0
	Yes	3.0
Other public places	No	8.0
	Yes	2.5
Garage	No	3.0
	Yes	1.5
Plant	No	8.0
	Yes	2.5
Warehouse	No	20.0
	Yes	4.0

#### Mesh division

The size of the grid affects the simulation accuracy. The coarser the mesh, the larger the data fluctuations which results in lower accuracy of the output results^13^. However, if the mesh is divided too small, it will affect the running time, and whether the CPU of the computer can drive the software is also another challenge. Therefore, selection of the appropriate mesh size is the first and most critical step in modeling.

According to the factor less expression D^*^ = *δ*_*x*_ given in Eq. 5 of the grid calculation in the operation manual, *δ*_*x*_ is the size of the grid cell. The formula is as follows^14^.

5 $$D^{*} = \left[ {\frac{Q}{{\rho_{0} c_{p} T_{0} \sqrt g }}} \right]^{{{2 \mathord{\left/ {\vphantom {2 5}} \right. \kern-0pt} 5}}}$$

where D* is the fire characteristic diameter, m; *Q* is the heat release rate of fire source, Kw; *g* is the gravitational acceleration, m/s^2^, taken as 9.8 m/s^2^; *C*_*p*_ is the constant pressure specific heat capacity, J/kg∙K, taken as 1.007 J/kg∙K; *ρ*_*0*_ is the air density, kg/m^2^, taken as 1.2 kg/m^2^; *T*_*0*_ is the ambient temperature, K, is taken as 293 K.

The fire source power selected in this paper contain 10 MW, and according to the formula, the resulting D = 2.4. In this case, the accuracy of the simulation results is higher when the ratio of fire feature value to grid size is between 4  and  16, so the grid takes the value size between 0.15 and 0.6, and the grid takes 0.5 × 0.5 × 0.5 considering the computer CPU and the computing time problem.

#### Fire conditions setting

**Figure 2:**
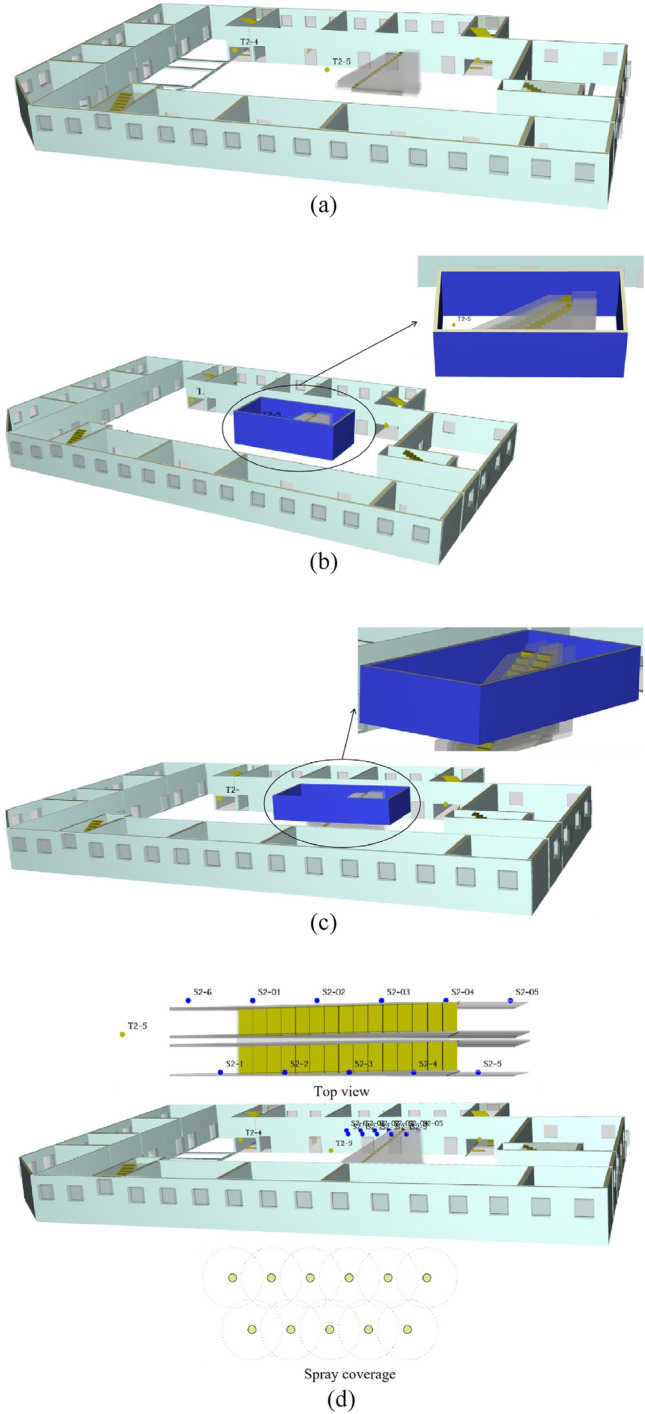
Diagram of each case of fire shutter. Notes: (**a**) represents the state of fire shutter corresponding to case 1; (**b**) represents the state of fire shutter corresponding to case 2; (**c**) represents the state of fire shutter corresponding to case 3; (**d**) represents the state of fire shutter corresponding to case 4.

The fire source is set on the floor 1, and the location of the fire source is shown in Fig. 1. In this paper, the changes of smoke transport, temperature, CO concentration and visibility in the mall under four working conditions of the escalator protective shutter were simulated. The four conditions are fire shutter failure, fire shutter normal, fire shutter down to 1.8 m from the ground, and setting sprinkler. The conditions design as shown in Table 3 and Fig. 2. Furthermore, temperature, CO concentration and visibility slices were set at 1.8 m on each floor, and temperature, the CO concentration and visibility detectors were set at each evacuation stairway entrance and escalator. The location of each detector is shown in Fig. 1. And it is clear from the fire regulations that the fire shutter on the fire source floor does not need to be lowered. Therefore, all the fire shutters in the 1st floor do not need to be lowered. According to GB14102-2005, the descending speed of vertical roll fire shutter is 2–7.5 m/min. In this paper, the descending speed of the fire shutter is 4.8 m/min, so the fire shutter is completely descending after 60 s of the fire. In addition, sprinklers of case 4 are turned on 60 s after the fire starts.

**Table 3:** Design of each fire condition.

Cases	Fire Source Location	Fire power	Fire shutter status
1	Floor 1	10 MW	Failure
Reference condition			Normal
3			Dropping to 1.8 m from the ground
4			Setting sprinkler(fire shutter not lowered)

#### Factors affecting personnel evacuation

Smoke poses the greatest threat to the life and safety of personnel in a building fire^14^. Therefore, when investing building fires, the impact of smoke to humans should be considered. Only a clear understanding and knowledge of the smoke generation process, development process, smoke transport laws and smoke types, and the different requirements and smoke evacuation codes, can a reasonable smoke evacuation system be designed to control building fire^15^. This will reduce the impact of fire smoke on evacuees. Failure to recognize the hazards of smoke during evacuation processes will result in fatal outcomes because combustible materials produce a large amount of toxic and high-temperature gases after burning. It is estimated that more than 80% of deaths due to fires are smoke-related.

1. Smoke temperature

Smoke exposes evacuees to high temperature, and the critical value of body temperature that the human body can withstand is 43–48 °C^16^. If the temperature of smoke above the human body is higher than 110 °C or the temperature of smoke below the head is higher than 60 °C, the human body temperature will increase to 43 °C cause fatal injury^17^. The effects of smoke temperature on human body under fire conditions are shown in Table 4.

**Table 4:** Effects of smoke temperature on human body.

Flue gas temperature (℃)	Effects on the human body
60	Begin to have adverse reactions
120	physical discomfort, can only last 10–15 min
140	Extreme discomfort, only lasted 5 min
170	May be fatal in 1 min

(2) CO concentration

Combustion under different fire types will produce a large amount of CO gas. Inhaled CO will combine with hemoglobin to produce carboxyhemoglobin, which makes decreases the oxygen carrying capacity of hemoglobin^18^. The effect of CO concentration on the human body is shown in Table 5.

**Table 5:** Effects of CO concentration in the inhaled gas on personnel.

CO concentration in the inhaled gas (kg·m^−3^)	Effects on the human body
2 × 10^–3^	Headache in 20 min
1 × 10^–3^	Headache and nausea in 45 min
5 × 10^–4^	Forehead pain in 2 h
2.5 × 10^–4^	Life threatening after 3 h
6.25 × 10^–5^	The maximum amount allowed for adults

(3) Visibility

According to the Chinese Fire Protection Handbook, the American SFPE Handbook and related references, the area larger than 5 m^2^ is defined as large space, otherwise it is small space. Otherwise, it is a small space, and the visibility threshold values of different spaces [53]–[54] are shown in Table 6.

**Table 6:** Visibility thresholds for different spaces.

Location	Small spaces	Large space
Visibility critical value/m	5 m	10 m

In accordance with Tables 4, 5 and 6 and the relevant provisions, the critical values of CO concentration and temperature in the smoke during evacuation are 2.5 × 10^–4^ kg·m^−3^ and 60 °C, respectively. The mall is a large space and the critical value for evacuation is 10 m.

### Personnel evacuation simulation

#### Introduction to Anylogic software

Anylogic software was developed by the Russian company XJ Technologies. It is used to conduct evacuation simulation based on the social force model and can provide three simulation modeling methods: multi-intelligent body, discrete event system, and system dynamics. Meanwhile, the Anylogic software is a relatively new pedestrian simulation software. Compared with the traditional simulation software, Anylogic software is closer to the real pedestrian characteristics. The software can visually count the time and number of people passing through a facility, and can update the density and distribution of people at any given time.

#### Shopping mall environment modeling and pedestrian flowchart construction

The Anylogic pedestrian library includes wall, rectangle area, target line, escalator group, among other parameters. It can create each floor with level layer, draw the outer boundary and inner wall of the mall with wall, and indicate each evacuation exit with target line. It uses the rectangle area and rectangle to build the stairs, as well as the escalator with escalator group to build the 3D physical model.

**Figure 3:**
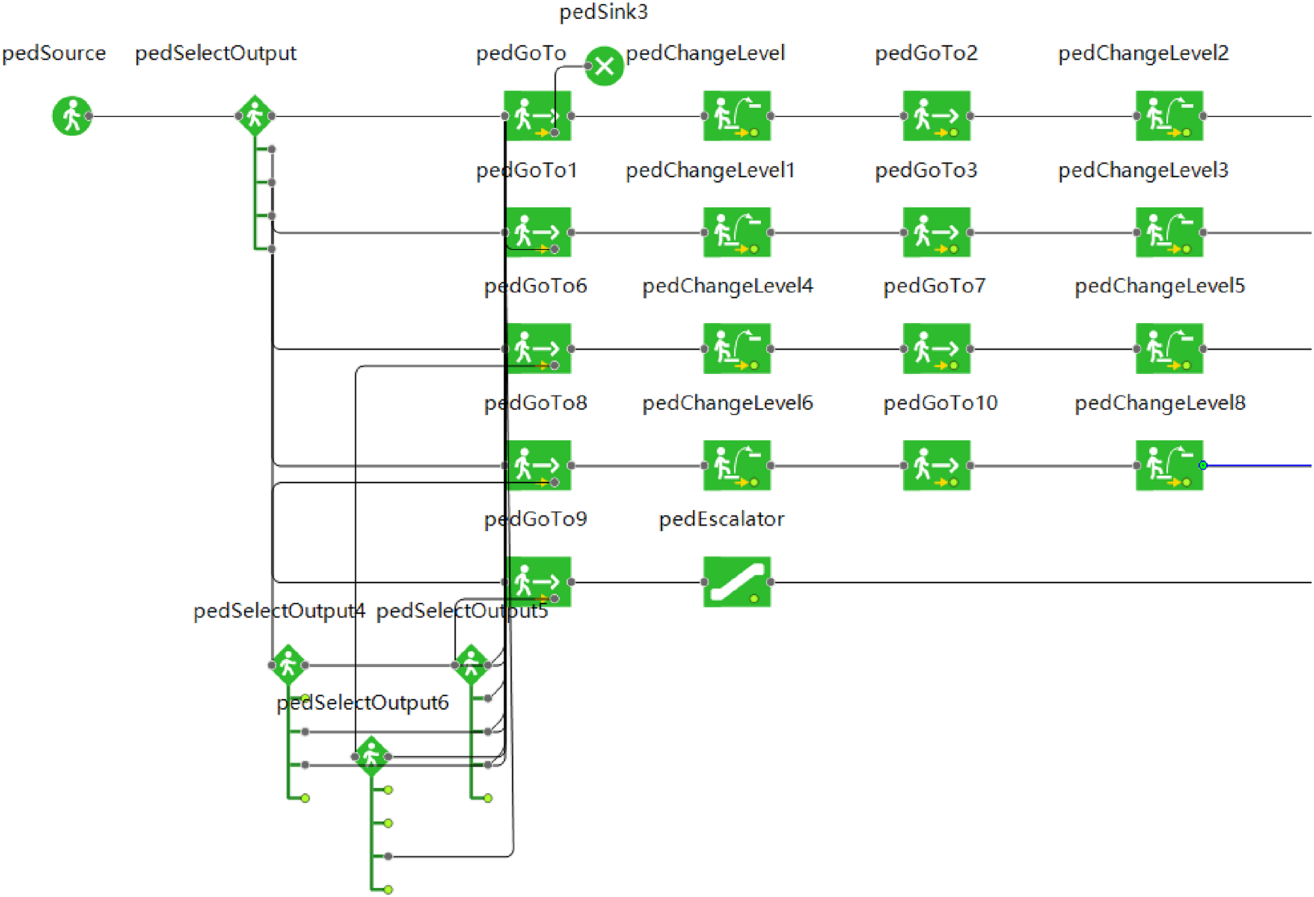
The floor 4 evacuation flow chart in case 3.

The personnel evacuation process is built through the pedestrian library as well as the process library using the environment model. By setting the PedSelectOutput, personnel are given priority to select the closest staircase or escalator. The events are set so that when the evacuation staircase or escalator at each level reaches the available safe evacuation time, personnel are prohibited from passing and are allowed to select other closer safe exits^14^. And when personnel use the escalator for evacuation, the escalator maintains its original operating status. When the model starts running, the constructed visual data window records the number of evacuees and the evacuation time. Some of the completed logic is shown in Fig. 3.

#### Basic parameter setting

The selected shopping mall has four floors. According to the Comprehensive Fire Code for Commercial Buildings, the number of evacuees per floor can be calculated based on the area of the mall's business hall. The specific calculation formula is as follows:

6 $$P = S_{y} \cdot \rho_{p}$$

where, *P* is the number of evacuees, Person; *S*_*y*_ is the area of shopping malls' business halls, m^2^; *ρ*_*p*_ is the personnel density, Person/m^2^.The value of *ρ*_*p*_ is taken through Table 7.

**Table 7:** Personnel density table.

Floor Position	Second basement level	First basement level	First and second floors above ground	Third floor on the ground	Fourth floor above ground and all floors above
Personnel density	0.56	0.6	0.43–0.60	0.39–0.54	0.30–0.42

**Figure 4:**
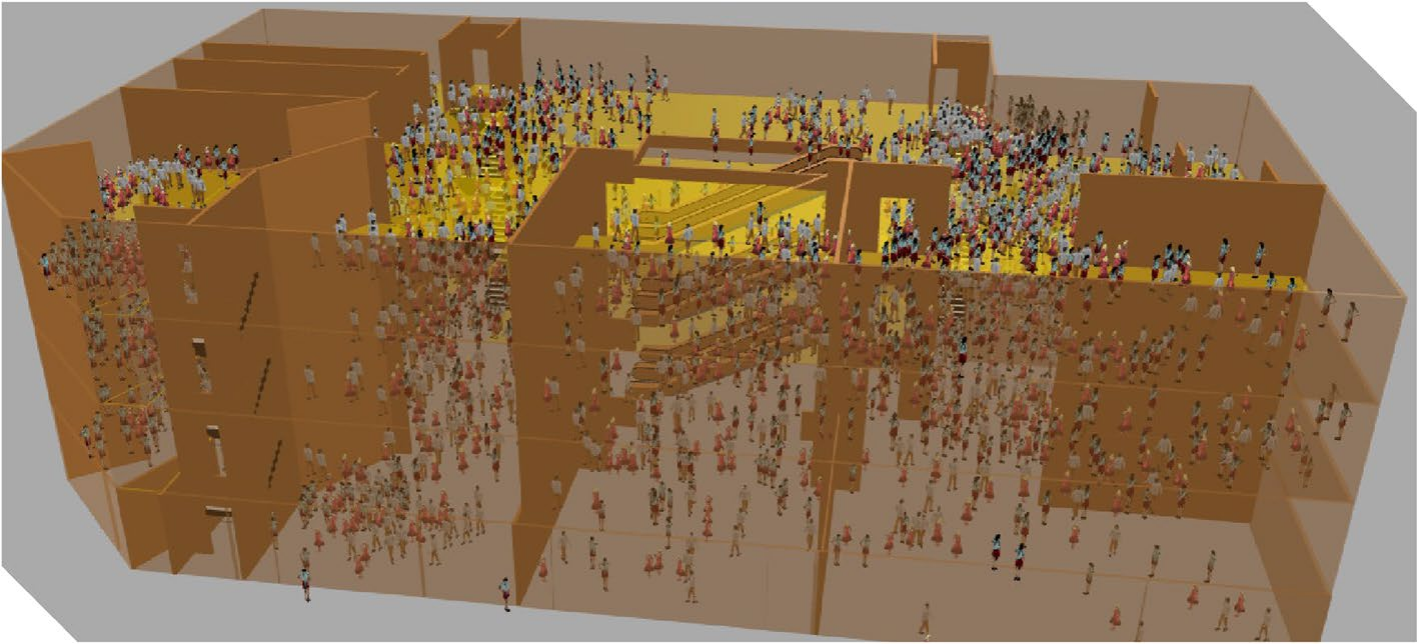
Map of initial positions of personnel.

When the area of the business hall is less than 3000 m^2^, the staff density goes to the upper limit. The area of the sales hall on the first floor of the mall is 600 m^2^, and the area of the sales halls on the second, third and fourth floors is 487 m^2^.So the personnel settings for each floor is shown in Table 8. The total number of people is 1118. The entire mall personnel load is evenly arranged. The initial location map of the mall personnel is shown in Fig. 4.

**Table 8:** Personnel load on each floor.

Floor	1F	2F	3F	4F
Personnel load/person	360	292	262	204

The number of evacuees, the speed of pedestrians and the shoulder width are set in the Pedsource module. According to previous studies and survey data, the evacuation personnel usually divides the crowd into: children, females, males and elderly. The proportion of the crowd is determined by combining the mall crowd distribution in the table. The pedestrian speed is divided into initial speed and desired speed in the Anylogic software, and the initial speed is based on the initial walking speed in the software. The pedestrian speed is influenced by factors such as gender and age, resulting in different expected pedestrian speeds. According to literature, the velocities of various types of pedestrians are determined as shown in the Table 9.

**Table 9:** Parameters of mall personnel.

Personnel category	Children	Females	Males	Elderly
Evacuation speed/(m/s)	1.1	1.3	1.33	0.9
Shoulder width/cm	27	38.7	41.5	30.8
Proportion/%	10	40	40	10

## Results analysis

### Fire scenario analysis

#### Smoke transport analysis

**Figure 5:**
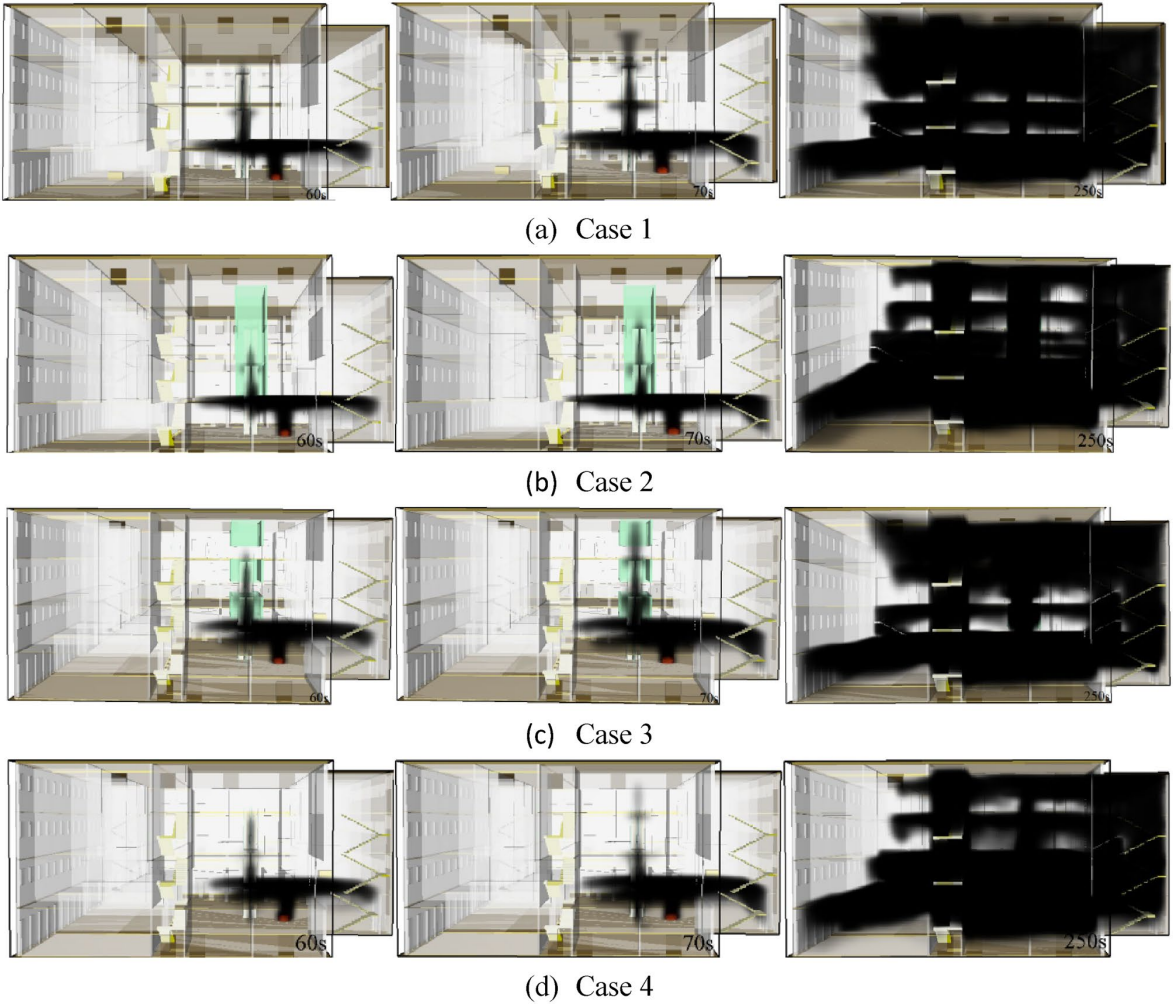
Flue gas cloud at t = 60 s, 70 s and 250 s for each case.

Data shown in the Fig. 5 indicate that the smoke of under all working conditions causes the chimney effect at the escalator. Figure 5a shows that the fumes of case 1 spread first to the top of the top floor, and the horizontal spread occurs at the escalator on the floor 2 at 60 s. The smoke of case 1 touches the top at 70 s, while spreading horizontally at the escalator on the floor 3. Due to the rapid spread of smoke through the stairs and escalators, the middle area of the 1st and 4th floors was covered with smoke at 250 s, and there are only a few areas on the second and third floors that are not covered by smoke. Figure 5b shows that, after the chimney effect occurs in case 2, the smoke spreads to the top of the floor 3 at 70 s. and smoke spreads and accumulates in the 1st and 2nd floor as the smoke spread in the stairwell. Compared to case 1, case 2 has less flue gas in floors 3 and 4. Figure 5c indicates that the smoke at the escalator of working condition 3 reaches the top of the floor 4 at 70 s. Subsequently, the smoke sinks and the sunken smoke is transported to other areas through the gap. The smoke at the escalator before 70 s does not diffuse horizontally. The smoke in the middle area of floors 2 and 3 is less than that in cases 1 and 2. However, the smoke of floor 4 is fully covered by smoke. According to Fig. 5d, the smoke at the escalator on the floors 3 and 4 was thin at 70 s. At 250 s, the area of the 1st and 2nd floors was full of smoke. And 3 and 4 layers are less smoky than case 1.

Notably, the area of flue gas spread is smallest under case 3 at 250 s. The smoke under cases 1 and 3 is mainly concentrated in the 1st and 4th floors. Since the clearance on the case 3 escalator is less than the case 1, and thus the smoke on the 2nd and 3rd floors is less than in case 1. The cases 2 and 4 are mainly concentrated in the 1st and 2nd floors. In case 2, smoke from other floors spreads through the stairs mainly due to the fall of the fire shutter, which results in less smoke in the middle area of the 3rd and 4th floors. Case 4 is mainly because water curtain is set at the location of fire shutter, which reduce the temperature of smoke, and therefore prevent the smoke from moving upward, leading to less smoke on the 3rd and 4th floors.

#### Temperature analysis and discussions

**Figure 6:**
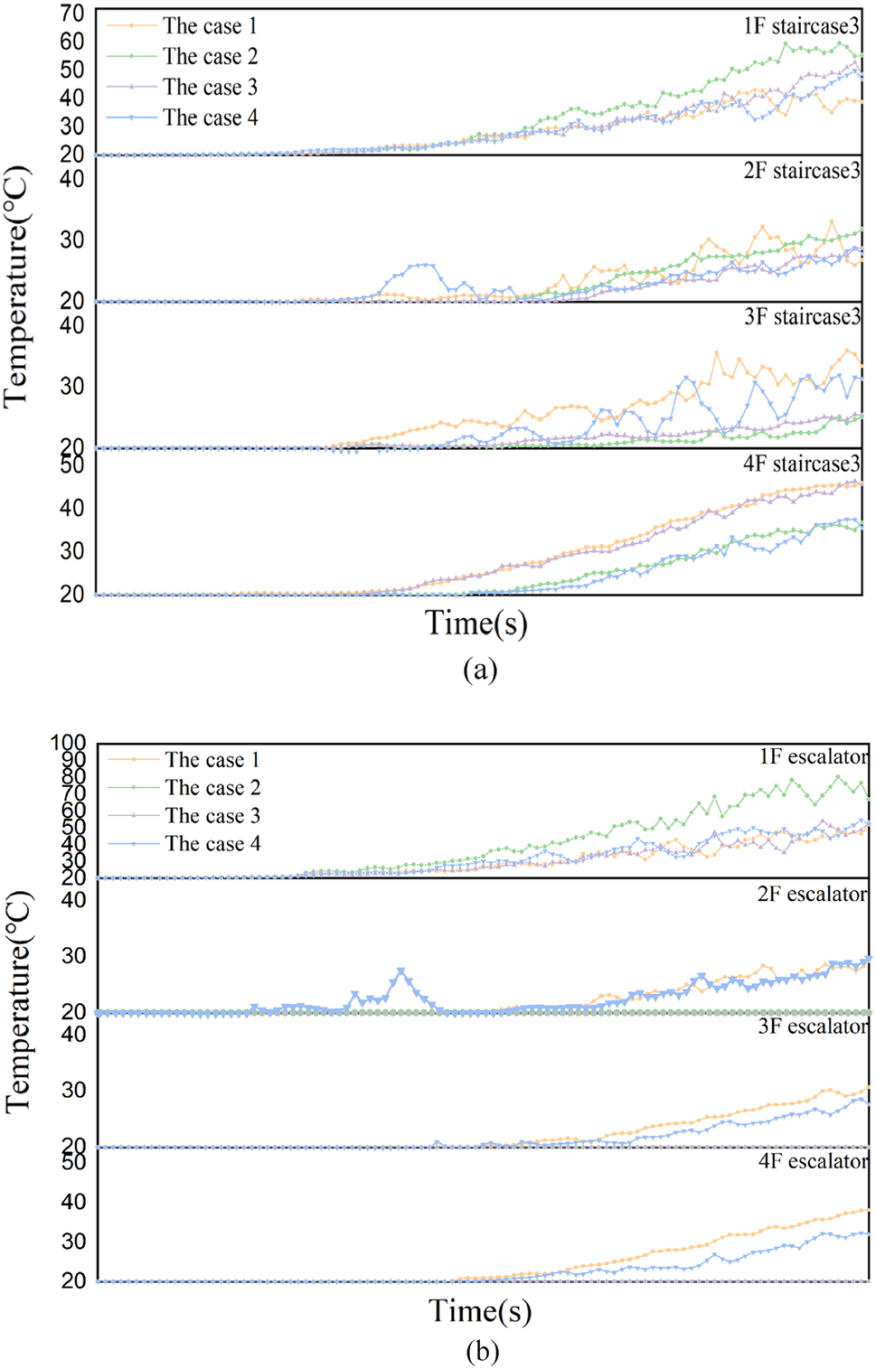
Temperature profiles at staircase 3 and the escalator for each working condition. (**a**) Temperature profiles at staircase 3. (**b**) Temperature profiles at the escalator.

Figure 6 shows the temperature curve at the evacuation staircase 3 and the escalator of each working condition. Notably, the floor 1 staircase 3 and the escalator of case 2 are at the highest temperature at the fire source layer. This mainly because that the fire shutter in the case 2 is completely down. When the smoke occurs at the escalator after the chimney, the smoke can not be discharged outside in time, the smoke at the escalator will accumulate a lot. For other working conditions, the state of the fire shutter at the escalator can transport the smoke to other floors, or the water curtain can reduce the smoke temperature. Therefore, the stairs 3 and the escalator of working condition 2 have the highest temperature at the fire source floor. Except for the fire source floor, the temperature of staircase 3 on floors 2 and 3 and escalators on floors 2, 3 and 4 were within 40 °C and the temperature of condition 1 was the highest. The highest temperature of the staircase 3 on floor 4 reached about 45 °C and the temperature of working conditions 1 and 3 was higher than that of working case 1 and 4.

The staircase 3 and automatic analysis of each floor revealed that the working conditions 1, 3 and 4 can reduce the temperature of the floor where the fire source is located and the temperature is decreased below the critical value for evacuation of people. However, other floors have the highest temperature under working condition 1. And except for the fire floor, the temperature at the escalator is at the initial temperature in working condition 3 and 4.

#### CO concentration analysis and discussions

**Figure 7:**
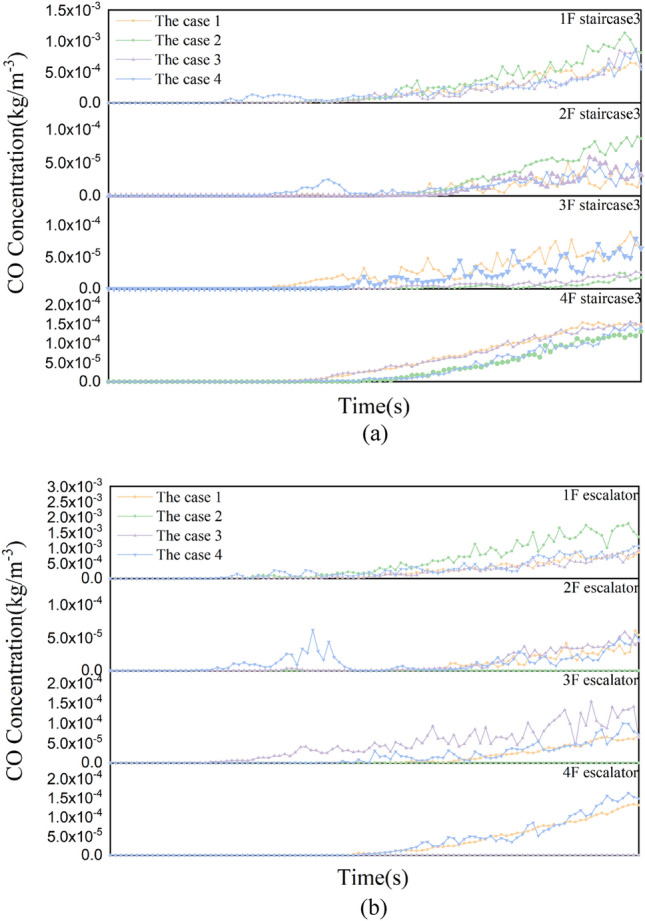
CO concentration curves at staircase 3 and the escalator for each working condition. (**a**) CO concentration curves at staircase 3. (**b**) CO concentration curves at the escalator.

Figure 7 shows that the CO concentration curves at evacuation stairs 3 and escalators for each working condition. The CO concentration at staircase 3 and the escalator on the fire source floor is similar to the temperature, and its concentration is highest at working condition 2. The CO concentration at staircase 3 on other floors was the highest in working condition 1. And escalators at 2^nd^ and 3^rd^ floors in working condition 3 concentration is the largest, and 4^th^ floor in working condition 1 concentration is the largest. However, except for the fire source floor, the CO concentration on other floors hardly affects the evacuation of people.

#### Visibility analysis and discussions

**Figure 8:**
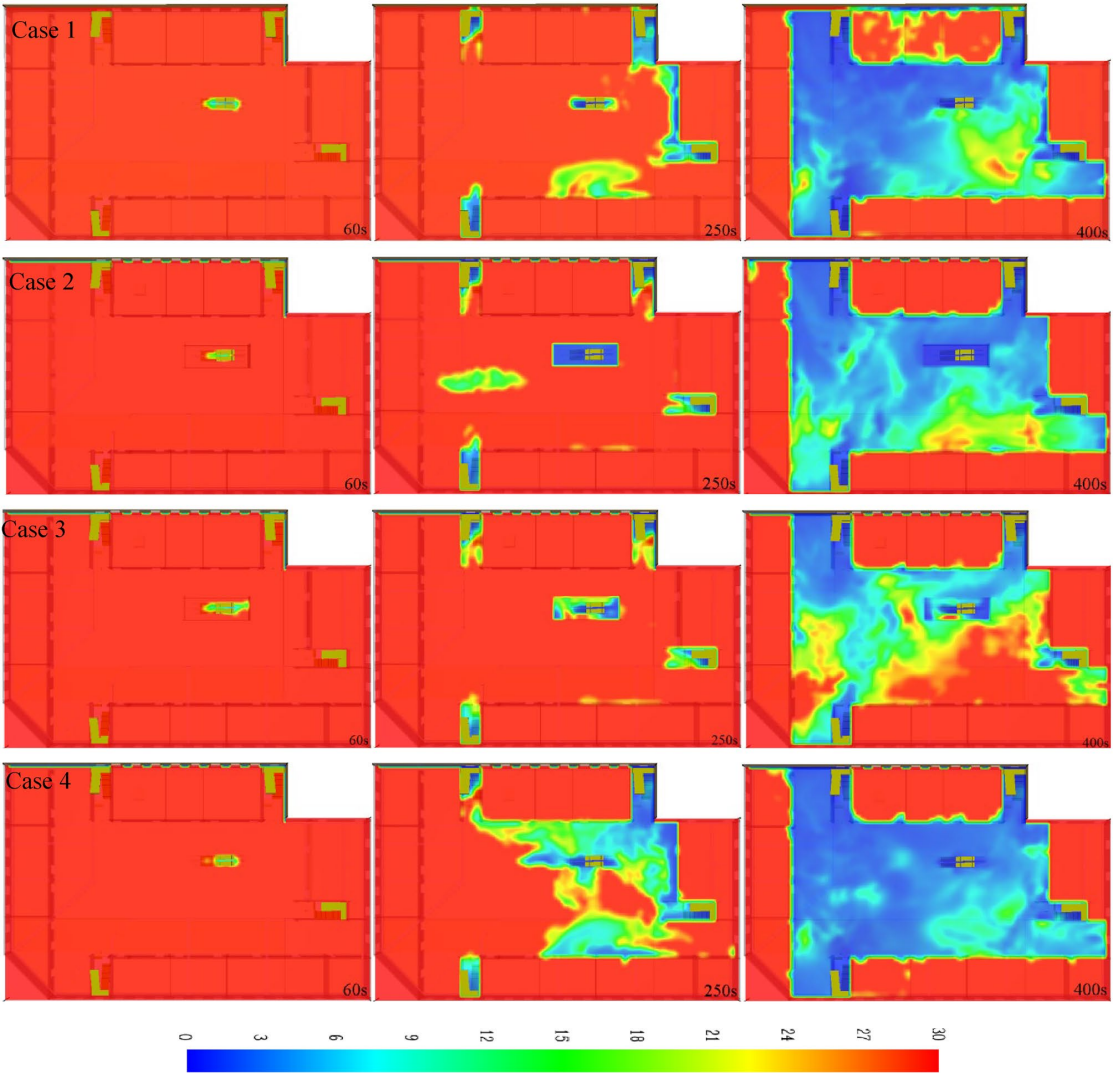
Visibility clouds at t = 60 s, 250 s, and 400 s for each working condition.

The visibility clouds at 2 m on the 2nd floor of each working condition are shown in Fig. 8. At 60 s, smoke accumulation at the escalator is more in at working condition and the visibility is less than 24 m. At 250 s, the visibility at each staircase and escalator of working condition 1 is below 10 m, and the visibility at staircase 1 and staircase 4 is similar in the four working conditions. The visibility near stairs 2 and 3 is lower compared with the other two conditions under working condition 1 and 4. The visibility at the escalator was below 10 m in the whole area inside the fire shutter in working condition 2, and the area with visibility below 10 m was the smallest in working condition 1. At 400 s, except for the left side of staircase 2 and the lower right corner of the escalator, the visibility in the middle area of working condition 1 below 10 m. The area visibility of working condition 2 is larger than that in the middle area of working condition 1 which is greater than 10 m. Case 3 has the smallest area with visibility less than 10 m, and some areas have visibility of 30 m. The visibility in the middle area of case 4 is nearly less than 10 m.

Analysis of the smoke migration in each working condition, the temperature, CO concentration, and visibility at the stairs and escalators, we infer that the fire shutter failure, fire shutter drop to 1.8 m from the ground, and setting sprinkler help to reduce the smoke hazard on the fire source floor. However, fire shutter failure will accelerate the spread of smoke, thereby increase the temperature and CO concentration on floors except the fire source floor, and reduce visibility. Compared with the fire shutter failure and setting sprinkler, the fire shutter down to 1.8 m from the ground, not only reduces the fire hazard to the floor but also ensures that the temperature and CO concentration at the stairs is suitable to evacuation, and the area with visibility less than 10 is the smallest. When the setting sprinklers, the temperature at the 4-level staircase and 3-level escalator, 4-story staircase CO concentration is less than the fire shutter down to 1.8 m from the ground. But the floors 3 and 4 temperature, and the CO concentration do not affect evacuation of people. Moreover, the area with visibility less than 10 m is the largest in setting sprinkler condition. Therefore, the fire shutter falling to 1.8 m from the ground is the most favorable condition for people evacuation.

### Analysis and discussion of available safe evacuation time for different working conditions

To analyze the available evacuation time for different working conditions, it is necessary to combine the tolerance of human body to fire products and simulate the developmental trend and law of combustion products such as carbon oxides, smoke and temperature of evacuation stairs and escalators on different floors of the building. The time required to reach the critical value of fire products can be considered as the available evacuation time for different passages.

**Figure 9:**
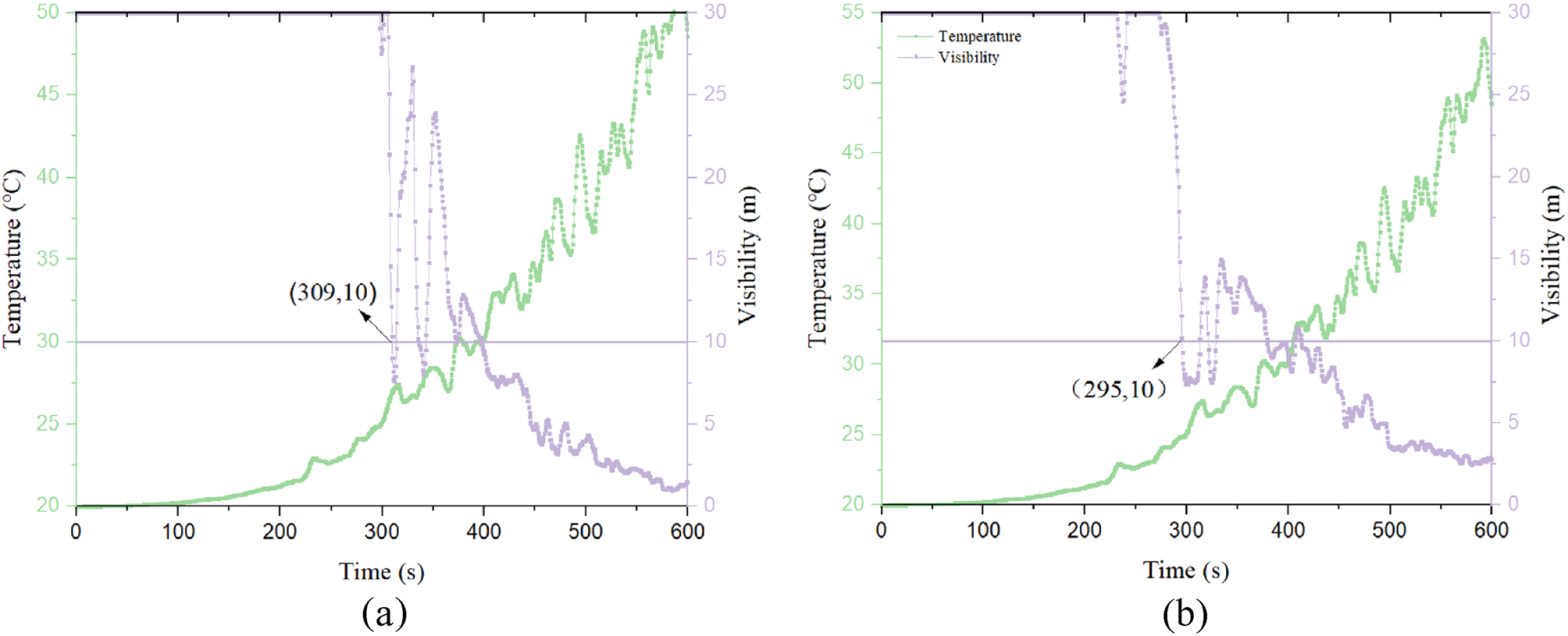
Temperature versus visibility over time. (**a** Case 3, **b** case 4 ).

**Figure 10:**
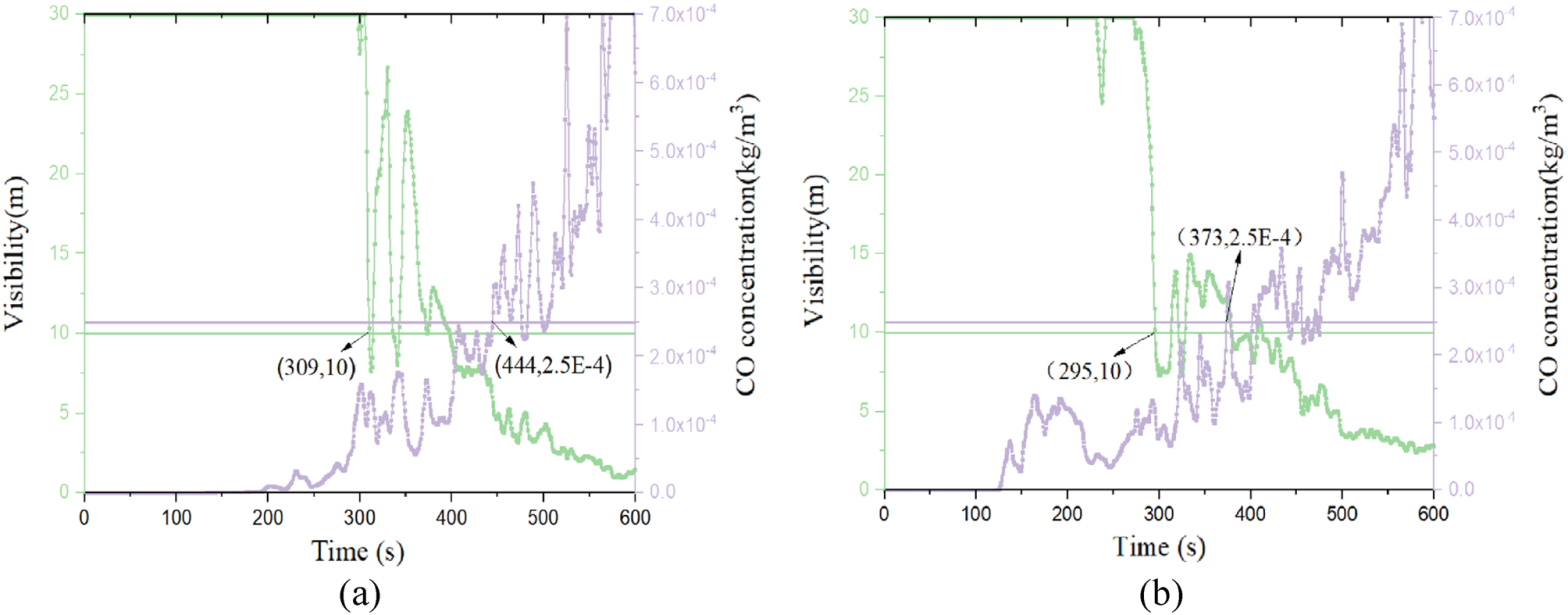
CO concentration versus visibility over time. (**a** Case 3, **b** case 4 ).

Based on the analysis of the temperature, carbon monoxide concentration, and visibility of the staircase3 and escalator for different operating conditions, it can be seen that both the evacuation staircase and escalator on the first floor are affected by the combination of temperature, carbon monoxide, and visibility to evacuate people. In contrast, the stairs and escalators on floors 2, 3 and 4 were mainly affected by visibility. Therefore, by analyzing the relationship between temperature increase time and visibility change, and the relationship between CO concentration and visibility. The graph of change relationship of case 3 and 4 Staircase 3 are shown in Figs. 9 and 10.

From Fig. 9, it can be seen that the visibility of working condition 3 reaches the critical value at 309 s, and working condition 4 reaches the critical value at 295 s. And the temperature in both conditions does not affect the personnel. According to Fig. 10, it can be seen that visibility is still the first to reach the critical value compared to carbon monoxide concentration. Therefore, the available safe evacuation time for the 1st floor staircase 3 is 309 s and 295 s for Case 3 and 4, respectively. The available safe time for other staircases and escalators and other cases for Case 3 and 4 are shown in Table 10.

**Table 10:** Available safe evacuation time for each staircase and escalator (s).

Fire conditions	Floor	Available safe evacuation time (s)				
		Staircase 1	Staircase 2	Staircase 3	Staircase 4	Escalator
Case 1	1st floor	297	295	324	139	318
	2nd floor	343	375	359	323	383
	3rd floor	322	252	210	370	393
	4th floor	279	253	239	266	321
Case 2	1st floor	294	331	369	279	–
	2nd floor	387	378	372	352	–
	3rd floor	322	427	541	428	–
	4th floor	356	265	340	399	–
Case 3	1st floor	298	300	309	345	308
	2nd floor	475	457	391	343	410
	3rd floor	385	267	390	454	160
	4th floor	331	249	242	237	117
Case 4	1st floor	302	335	295	339	115
	2nd floor	302	210	212	331	136
	3rd floor	389	239	278	368	278
	4th floor	371	268	344	377	330

**Figure 11:**
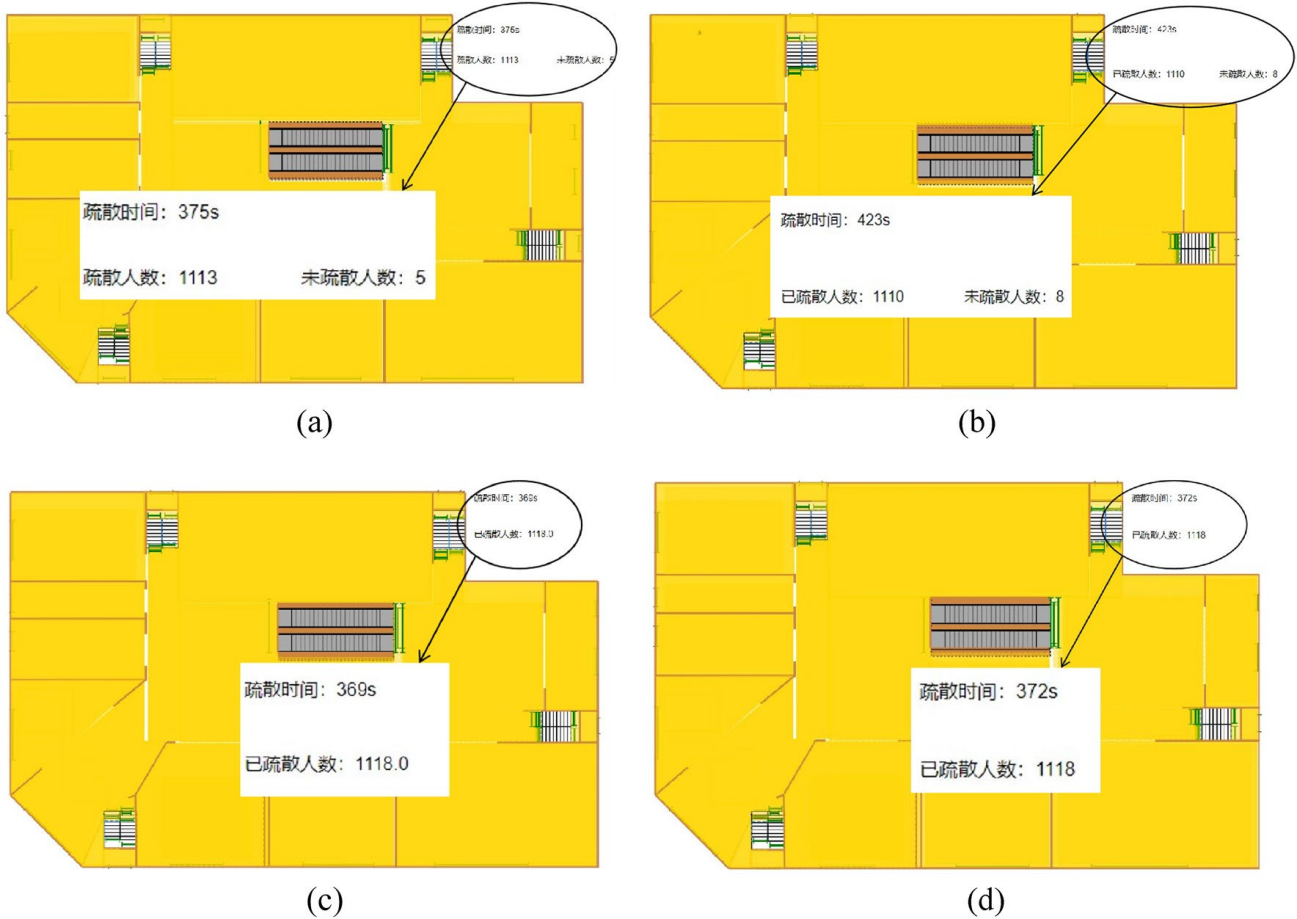
Simulation diagram at the end of each working condition. (**a** case 1; **b** case 2; **c** case 3, **d** case 4).

**Figure 12:**
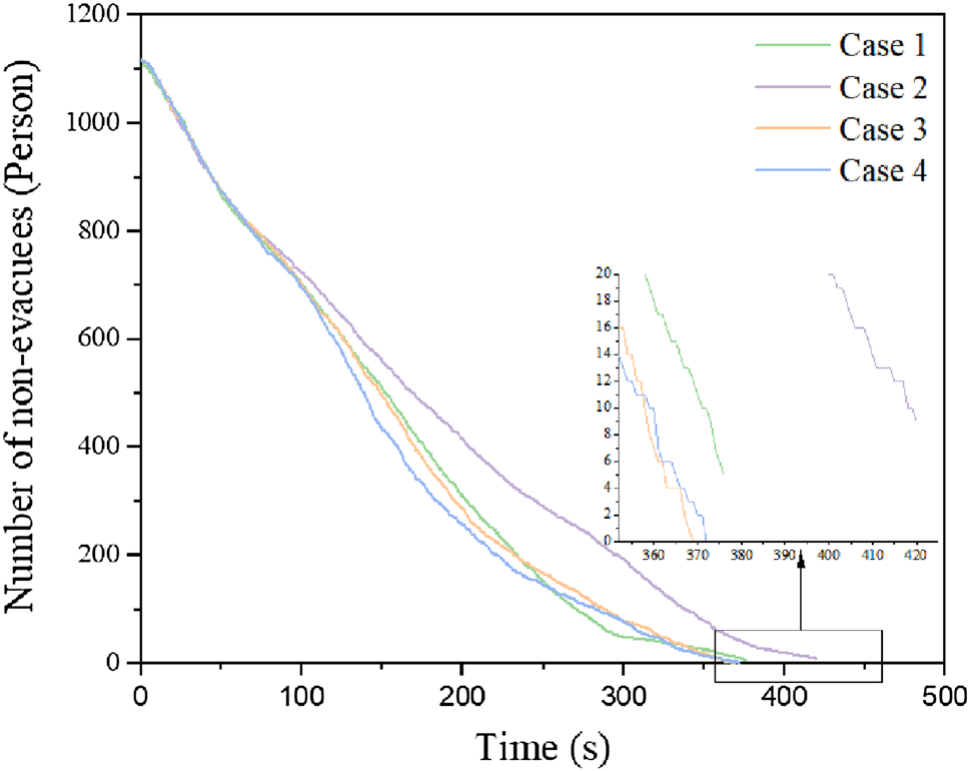
A curve showing changes in the number of unevacuated persons in each working condition with time.

Using the available safe evacuation time for each staircase and evacuation staircase obtained from the above Pyrosim simulation results of fire under different working conditions, each evacuation staircase and escalator is controlled in the Anylogic software by setting time events regardless of whether they are normally used for evacuation or not. When the set time event occurs, the personnel will abandon this passage and choose other safe evacuation path. In this paper, when the escalator is used for evacuation, it is stopped. The simulations at the end of each condition are shown in Fig. 11.The total evacuation time versus the number of people not evacuated curve is shown in Fig. 12.

According to Figs. 11 and 12, under fire conditions, the number of unevacuated persons in each working condition decreases with similar patterns before 80 s. After 80 s, the number of unevacuated persons in each working condition gradually decreases at a smaller rate. Between 80 and 236 s, the number of unevacuated persons in working condition 4 is the least, whereas that in working condition 2 is the highest. At the end of evacuation, the number of unevacuated persons in working condition 1 and working condition 2 were 5 and 8, respectively, within the available safe evacuation time. In working conditions 3 and 4, the evacuation was completed within the available safe evacuation time, and the evacuation time was 369 s and 372 s. In working condition 1, the fire shutter failed and people evacuated by escalator within the available safe evacuation time, but the fire developed rapidly due to the lack of smoke control measures, making it unsuitable to evacuate people than working condition 2. In working condition 2, the fire shutter is normal and the personnel can only evacuate through 4 staircases. Moreover, there are fewer evacuation passages than working condition 3 and 4, hence the personnel are easily crowded at the staircases. As a result that 8 persons does not complete the evacuation.

## Discussions

A design strategy for improving personnel evacuation efficiency for escalator evacuation is proposed. This strategy is demonstrated in a case study, where escalator evacuation from a shopping mall is studied. The case study illustrates the effect of different operating conditions of fire shutters at escalators on the evacuation of people and obtains the favorable operating conditions of fire shutters for evacuation of people. Therefor, this study found that the shortest evacuation time for people using the escalator in a fire scenario was when the fire shutter at the escalator was lowered to 1.8 m from the ground.

Based on previous studies, scholars have performed some numerical simulations of evacuation^7^. However, there is no analysis of escalator evacuation in the fire source environment. And the effect of the state of the escalator's fire shutter on evacuation has not been studied. Therefore, this study takes the fire evacuation of a shopping mall as the research object. First, we simulated the effect of different working conditions of the fire shutter at the escalator on the smoke from a mall fire and derived the available safe evacuation time for personnel evacuation under different working conditions. Then, the evacuation simulation of personnel was carried out on the basis of the available safe evacuation time for different working conditions. The available safe evacuation times for different working conditions were obtained. To this end, this paper proposes the evacuation of people using escalators in a fire environment. A new evacuation route can be provided for buildings with escalators. In addition, the existing escalator evacuation can only determine the improvement of personnel evacuation efficiency, but cannot guarantee the safety of personnel evacuation. Therefore, we also studied the effect of the fire shutter at the escalator down to 1.8 m from the ground and the additional sprinklers on the evacuation of personnel. As can be seen from Fig. 8, the fire shutter failed (or not fall) and the fire shutter fell normally with 2 people and 1 person not completing evacuation respectively, and the evacuation time was longer. And when the fire shutter fell to the ground 1.8 m or additional sprinkler evacuation time was shortened, and all people completed evacuation. Therefore, in the process of evacuation of personnel using escalators, the escalator fire shutter should be considered to drop to 1.8 m from the ground or additional sprinklers. Compared the water curtain setted, the evacuation time is shorter when the fire shutter is lowered to 1.8 m from the ground. Moreover, when the fire shutter is lowered to 1.8 m from the ground, the average available safe evacuation time of the stairs on each floor is longer, and the evacuation of people is more utilized. Therefore, the fire shutter lowered to 1.8 m from the ground utilizes the evacuation of people more than the water curtain, and the evacuation efficiency is higher.

In conjunction with the case study, several knowledge gaps in the areas of escalator evacuation was identified. Specific areas that need to be investigated further include (1) How to lower the escalator to 1.8 m from the ground, (2) How to guide people to evacuate using escalators and (3) escalator loading. Laboratory and field experiments are needed to further explore these areas.

## Conclusions

1. The analysis of smoke migration, temperature, CO concentration and visibility under the fire shutter failure, normal, drop to 1.8 m from the ground and setting sprinkler at the escalator of the shopping mall shows that the temperature and CO concentration of the fire source floor have a significant impact on evacuation of personnel, while evacuation of people on other floors is mainly affected by visibility.
2. Simulation of personnel evacuation under different fire conditions using Anylogic software showed that there was incomplete evacuation of personnel when the mall was crowded under fire shutter failure or normal. In the fire shutter down to 1.8 m from the ground or the use of setting sprinkler can reduce the evacuation time, but the fire shutter down to 1.8 m from the ground is more suitable to the evacuation of personnel.
3. Analysis of the evacuation of the mall fire scenario shows that the mall has evacuation channels that do not meet the personnel evacuation needs. Installation of escalators in malls can ease the overload of evacuation stairs. In addition, fire training and professional command systems should be put in place to ensure orderly evacuation of personnel in case of fire.
4. In this paper, numerical simulations of fire and safe emergency evacuation of a shopping mall were conducted using Pyrosim and Anylogic software. The smoke dispersion law, smoke concentration, temperature, visibility and personnel evacuation law under fire condition were analyzed and studied. This paper is to determine whether escalators can be used for evacuation studies and the focus is how they can be used optimally to ensure safe evacuation. These research results provide a new safe evacuation mode for mall in case of fire.

## Data Availability

The data used to support the findings of this study are available from the corresponding author upon request.

## References

[1] Lama, S. & Mishra, A. K. Operational assessment of fire safety status of existing commercial buildings at Birtamode, Jhapa, Nepal (2022).

[2] Shoshe MAMS, Rahman MA (2022). Improvement of heat and smoke confinement using air curtains in informal shopping malls. J. Build. Eng..

[3] Jia J, Tian X, Wang F (2022). Research on smoke control for an underground mall fire, based on smoke barrier and mechanical smoke exhaust system. Sci. Rep..

[4] Sun SL, Zhao Q, Xie WZ (2020). Study on safe evacuation routes based on crowd density map of shopping mall. IEEE Access.

[5] Han, F., Liu, L. & Zhang, Y. Pathfinder-based simulation and optimisation of personnel evacuation modelling of a shopping mall. In *Journal of Physics: Conference Series* Vol. 1757, 012112 (IOP Publishing, 2021).

[6] Huang Z (2010). The auxiliary role of escalators and moving sidewalks in safe evacuation. J. Nanchang Coll..

[7] Naoko K, Hasemi Y, Moriyama S (2012). Feasibility of upward evacuation by escalator—An experimental study. Fire Mater..

[8] Hiroyuki K, Sekizawa A, Takahashi W (2012). Study on availability and issues of evacuation using stopped escalators in a subway station. Fire Mater..

[9] Tang, J. L. Study on fire evacuation optimization planning of large commercial buildings. Dalian University of Technology (2020).

[10] Ma H, Zhang HB, Zhu HJ (2021). Study on multi-channel coupling evacuation strategy of stairs, escalators and elevators in commercial complex. J. Saf. Sci. Technol..

[11] Wang JY, Zong RW, Lu SX (2021). Dynamic evaluation of consequences of toxic gas dispersion in fire of crowded places. China Saf. Sci. J..

[12] Hasnain, S. A., Nasif, M. S. & Pao, W. et al. Numerical investigation of smoke contamination in atrium upper balconies at different down stand depths. In *Building Simulation* Vol. 10, 365–381 (Tsinghua University Press, 2017).

[13] Guo-qing Z (2022). A case study on safety evacuation of large covered mall with common pedestrian area. Fire Sci. Technol. (Beijing).

[14] Mishra K, Aithal PS (2022). Preparedness and costing on fire safety installation in commercial buildings. Int. J. Appl. Eng. Manag. Lett. (IJAEML).

[15] Rahman, N. V. & Nadapdap, M. H. Analysis of the effectiveness of evacuation paths in terms of mall visitors evacuation speed (Case Study: Mall Palladium). In *IOP Conference Series: Earth and Environmental Science* Vol. 452, 012093 (IOP Publishing, 2020).

[16] Zi-chao MA, Chao YAN, Jun MA (2021). Fire risk analysis and countermeasures of an underground wholesale shopping mall. Fire Sci. Technol..

[17] Datta S, Behzadan AH (2019). Modeling and simulation of large crowd evacuation in hazard-impacted environments. Adv. Comput. Des..

[18] Krajewski, G. & Węgrzyński, W. Use of computational fluid dynamics in optimization of natural smoke ventilation from a historical shopping mall–Case study. In *AIP Conference Proceedings* Vol. 1922, 110009 (AIP Publishing LLC, 2018).

